# Association of Genetic Variants with Primary Angle Closure Glaucoma in Two Different Populations

**DOI:** 10.1371/journal.pone.0067903

**Published:** 2013-06-28

**Authors:** Mona S. Awadalla, Suman S. Thapa, Alex W. Hewitt, Kathryn P. Burdon, Jamie E. Craig

**Affiliations:** 1 Department of Ophthalmology, Flinders University, Flinders Medical Centre Adelaide, South Australia, Australia; 2 Nepal Glaucoma Eye Clinics, Tilganga Institute of Ophthalmology, Kathmandu, Nepal; 3 Centre for Eye Research Australia, University of Melbourne, Royal Victorian Eye and Ear Hospital, Melbourne, Australia; Duke University, United States of America

## Abstract

**Purpose:**

A recent large genome-wide association study (GWAS) identified multiple variants associated with primary angle-closure glaucoma (PACG). The present study investigated the role of these variants in two cohorts with PACG recruited from Australia and Nepal.

**Method:**

Patients with PACG and appropriate controls were recruited from eye clinics in Australia (n = 232 cases and n = 288 controls) and Nepal (n = 106 cases and 204 controls). Single nucleotide polymorphisms (SNPs) rs3753841 (*COL11A1*), rs1015213 (located between *PCMTD1* and *ST18*), rs11024102 (*PLEKHA7*), and rs3788317 (*TXNRD2*) were selected and genotyped on the Sequenom. Analyses were conducted using PLINK and METAL.

**Results:**

After adjustment for age and sex, SNP rs3753841 was found to be significantly associated with PACG in the Australian cohort (p = 0.017; OR = 1.34). SNPs rs1015213 (p = 0.014; OR 2.35) and rs11024102 (p = 0.039; OR 1.43) were significantly associated with the disease development in the Nepalese cohort. None of these SNPs survived Bonferroni correction (p = 0.05/4 = 0.013). However, in the combined analysis, of both cohorts, rs3753841 and rs1015213 showed significant association with p-values of 0.009 and 0.004, respectively both surviving Bonferroni correction. SNP rs11024102 showed suggestive association with PACG (p-value 0.035) and no association was found with rs3788317.

**Conclusion:**

The present results support the initial GWAS findings, and confirm the SNP’s contribution to PACG. This is the first study to investigate these loci in both Australian Caucasian and Nepalese populations.

## Introduction

Primary angle-closure glaucoma (PACG) is a subtype of glaucoma characterised by obstruction of the irido-corneal angle, increase in the intraocular pressure, and slow progressive destruction of the optic nerve with corresponding loss of the peripheral visual field. [1] Glaucoma is the leading cause of irreversible blindness worldwide, with primary angle-closure glaucoma accounting for almost half of all blind glaucoma patients. [2] The number of patients with PACG is expected to rise by approximately 5 million people from 16 million over the next decade. [1].

Interestingly, affected individuals tend to exhibit a consistent spectrum of anatomical biometric features such as reduced anterior chamber depth along with narrowing in the irido-corneal drainage, increased lens thickness and change in its position, hyperopic refractive error, and short axial length. [3] The disease is more prevalent in older age groups, in females, and in certain populations including Eskimos and Asians. [4].

Primary angle-closure glaucoma is a complex heterogeneous disease, with the genetic susceptibility under investigation. Recently, a two staged genome wide association study (GWAS) was conducted on a large cohort with PACG (3,771 cases and 18,551 controls) from multiple ethnicities. Three susceptibility loci were detected at genome-wide significance on meta-analysis of all data from both stages; *PLEKHA7* rs11024102, *COL11A1* rs3753841, and rs1015213 located between *PCMTD1* and *ST18.* [5].

*PLEKHA7* (pleckstrin homology domain-containing family A member 7) located on Chromosome 11, is an adherens junction protein [6]. Adherens junction (AJ) are required for organization of the epithelial architecture [7], and contribute to tissue homoeostasis. [8] It is likely to be involved in affecting the fluid flow across the inner wall of Schlemm’s canal. [9] It was proposed that mutation in *PLEKHA7* could affect the fluid dynamics in the pathophysiology of angle-closure glaucoma. [10].

*COL11A1* encodes one of the two alpha chains of type Xl collagen. It is associated with type II Stickler and Marshall syndromes [11]–[13] which are congenital conditions that include high myopia and blindness from retinal detachment. [14] The GWAS data showed a single SNP, rs3753841, to be associated with PACG, and the authors proposed the causal variants predisposing towards PACG within *COL11A1* may alter its gene expression such as to engineer a reverse effect to that observed in myopic eyes.

The third locus, rs1015213, is located on chromosome 8 between two genes, *PCMTD1* and *ST18,* but as the associated was found to be in LD with *PCMTD1*, it was suggested as the most likely candidate gene. *PCMTD1* encodes protein-L-isoaspartate O-methyltransferase domain-containing protein 1. Very little is known about the function of this gene, but it is expressed in eye tissues including iris and trabecular meshwork which are involved in the pathogenesis of PACG. *PLEKHA7* and *COL11A1* are also found to be expressed in most eye tissues, especially in iris and trabecular meshwork.

The GWAS study reported a fourth locus rs3788317 that did not reach the level of genome wide significance in the meta-analysis. It is located in an intron of the *TXNRD2* (thioredoxin reductase 2) gene on chromosome 22. Thioredoxin reductase has been recently found in the lens and is reported to participate in the repair process of oxidative damaged lens proteins/enzymes in patients with cataracts. [15] The variations within this gene may participate in the pathogenesis of PACG, since one of the clinical risk factors of PACG is change in thickness and position of the lens.

The present study aimed to investigate the replication of these SNPs in our PACG cohort recruited from two populations from Australia and Nepal.

## Materials and Methods

Approval was obtained from the human research ethics committee of the Southern Adelaide Health Service and Flinders University, and the Institutional Review Committee of the Tilganga Institute of Ophthalmology. This study has been conducted in accordance with the Declaration of Helsinki and its subsequent revisions. Written informed consent was obtained from each individual.

Australian Caucasian participants were recruited from Ophthalmology clinics in Australia through the Australian and New Zealand Registry of Advanced Glaucoma ANZRAG. [16] All participants reported in this study were Caucasian. The Nepalese cohort was recruited from the Nepal Glaucoma Eye Clinic, Tilganga Institute of Ophthalmology, Kathmandu Nepal by one of the authors (S.S.T). [17], [18].

Each participant underwent a complete eye examination including; slit lamp examination of the anterior chamber, gonioscopy, best corrected visual acuity, measurement of intraocular pressure, fundus examination with special attention to optic disc parameters, and visual field assessment. Refraction was carried out using a streak retinoscope (Beta 200, Heine, Germany), followed by a subjective refraction. [17].

232 Australian and 106 Nepalese participants identified with PACG were recruited. Patients with primary angle-closure glaucoma were included using the definition of the International Society of Geographical and Epidemiological Ophthalmology (ISGEO) described by Foster and colleagues. [19] It was based on the presence of glaucomatous optic neuropathy with cup:disc ratio≥0.7, intraocular pressure greater than 21 mmHg, peripheral visual loss, presence of at least 180 degrees of closed angle in which the trabecular meshwork is not visible on gonioscopy.

Controls were required to have none of the above characteristics, and no family history of glaucoma or previous surgery for glaucoma. Participants with pseudophakia or secondary angle-closure glaucoma caused by events such as uveitis, trauma or lens subluxation were excluded. The control group consisted of 288 Australian and 204 Nepalese individuals. The Australian cohort was ascertained from retirement villages in Adelaide, South Australia. Nepalese controls were participants in a population-based study of Kathmandu, Nepal. Control individuals were chosen specifically to be matched for gender and ethnic group to the Nepalese cases. Controls from both cohorts were slightly older than cases by design for this aging disease.

Genomic DNA was extracted from peripheral whole blood using the QiaAmp Blood Midi (Nepalese samples) or Maxi (Australian samples) Kit (Qiagen, Valencia, California). SNPs were selected from the literature; rs3753841 (*COL11A1*_ NM_080630.3), rs1015213 (located between *PCMTD1* and *ST18*), rs11024102 (*PLEKHA7* NM_175058.4), and rs3788317 (*TXNRD2* NM_006440.3). [10] Genotyping was conducted using the iPLEX Gold chemistry (Sequenom Inc, San Diego, California) on an Autoflex mass spectrometer (Sequenom Inc, San Diego, California) at the Australia Genome Research Facility (AGRF), Brisbane.

Power calculations were conducted in each cohort to assess the power of the study to detect association at the four tested SNPs across a range of effect sizes from 1.0 to 2.4, using the minor allele frequency observed for each SNP in each cohort in this study. [20].

Association analyses were conducted using PLINK. [21]. Each cohort was analysed separately withadjustment for sex and age under an additive model using multivariate logistic regression. Combined analyses were conducted using the Cochran-Mantel-Haenszel 2×2×K test (cmh) performed to allow for differences between the populations. The differences in the odds ratios between the results of the current study and previously reported results were viewed in Forest Plot Viewer v1.00 which generates forest plots from odds ratio and confidence interval inputs. [22].

Meta-analysis of the two cohorts was conducted using the age and sex adjusted results, using logistic regression and inverse variance weights to calculate an overall z-statistics and p-value with the METAL program. [23].

## Results

Replication of reported PACG associated SNPs was investigated using independent cohorts with PACG from Australia and Nepal. We genotyped the top ranked SNPs from the previous reported GWAS study; rs3753841 (*COL11A1*), rs1015213 (located between *PCMTD1* and *ST18*), rs11024102 (*PLEKHA7*), and rs3788317 (*TXNRD2*).

**Figure 1:**
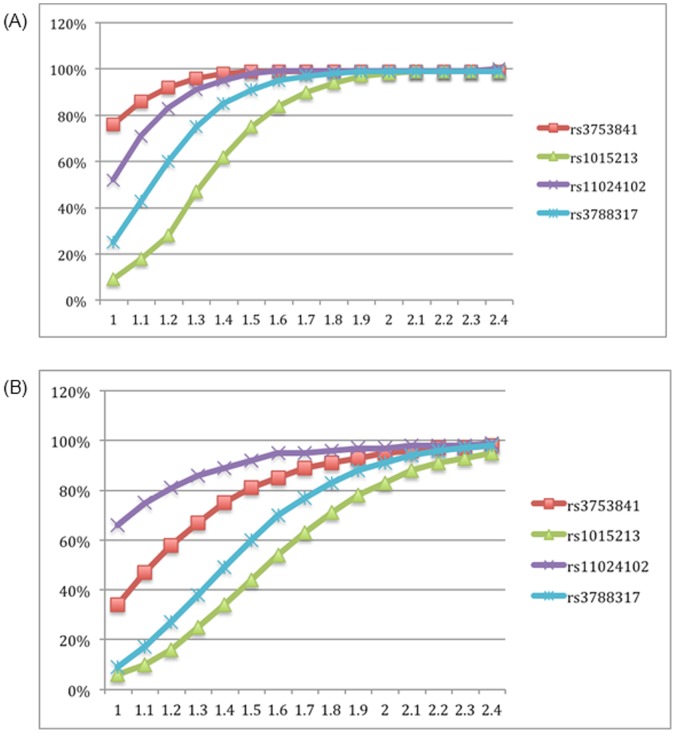
Plot showing the study power of the selected SNPs in both (A) Australian (n = 232 cases and n = 288 controls) and (B) Nepalese (n = 106 cases and 204 controls) populations per-allele odds ratio. X-axis represents relative risk (range from 1.0 to 2.4) and Y-axis the power in percentage. Minor allele frequencies of each SNP are presented in Table 1.

Power calculations were undertaken to determine if the current cohorts were sufficiently powered to detect genetic association with the disease at α = 0.05 level assuming complete linkage disequilibrium between the disease causing variant and the marker, in both cohorts. The overall prevalence of PACG is estimated to be 0.4% in European derived populations [24] and 0.43% in Nepalese population. [25] The power varies between SNPs and cohorts due to differences in the allele frequencies in each population and the size of each cohort. The Australian cohort has 80% power to detect an association at least as big as that previously reported at all SNPs except rs1015213. The Nepalese cohort can detect associations with confidence at both SNPs rs3753841 and rs11024102 (Figure 1).

The allele frequencies and associations were evaluated in each cohort independently with age and sex adjustment as shown in Table 1. In the Australian cohort, rs3753841 (*COL11A1*) was associated under the additive model (p = 0.017, OR 1.34; 95% CI 1.1 to 1.7). No association was observed in the Nepalese cohort for this SNP. Conversely, both rs1015213 (located between *PCMTD1* and *ST18*) and rs11024102 (*PLEKHA7*) were found to be significantly associated with PACG in the Nepalese cohort (p = 0.014 and p = 0.039, respectively). After applying Bonferroni correction for multiple testing in the population-specific analysis none of these SNPs showed significance.

**Table 1:** Minor allele frequencies (%) of SNPs, and p-value adjusted for age and sex for the Australian and Nepalese cohorts with the odds ratio (95% CI).

		Australian				Nepalese			
SNP	Minor Allele	MAF Case	MAF Control	p-value	OR (95% CI)	MAF Case	MAFControl	p-value	OR(95%CI)
rs3753841	G	0.43	0.36	0.017	1.34 (1.1–1.7)	0.35	0.31	0.308	1.20 (0.7–1.3)
rs1015213	A	0.12	0.09	0.157	1.41 (0.9–2.0)	0.10	0.05	0.014	2.35 (1.3–3.8)
rs11024102	G	0.31	0.29	0.411	1.10 (0.9–1.4)	0.50	0.41	0.039	1.43 (1.0–1.6)
rs3788317	A	0.22	0.22	0.75	1.03 (0.8–1.3)	0.16	0.17	0.742	0.92 (0.5–1.0)

P-value of less than 0.013 is considered significant. OR = Odds ratio, MAF = minor allele frequency.

The combined analysis (Table 2) using the Cochran-Mantel-Haenszel test, unadjusted for covariates, showed three SNPs to be significantly associated with PACG; rs3753841 with p-value of 0.009 (OR 1.31), rs1015213 with p-value of 0.004 (OR 1.61), and rs11024102 with p-value 0.035 (OR 1.25). After applying correction for multiple testing (Bonferroni), both rs3753841 and rs1015213 remained significant. When the meta-analysis was conducted using sex and age adjusted results, both rs3753841 (p-value = 0.011) and rs1015213 (p-value = 0.011) were significantly associated with the disease. By assessing the I^2^ index that measures the extent of heterogeneity between sample collections in the meta-analysis (<25% indicates low heterogeneity, 25%<I^2^<50% moderate heterogeneity, and >50% high heterogeneity), SNPs rs11024102 and rs1015213 had moderate heterogeneity (), which was not significant (Table 2). SNPs rs3753841 and rs3788317 showed no heterogeneity (Table 2).

**Table 2:** Association results of previously reported PACG SNPs after adjustment for population stratification under a Cocrhan-Mantel-Haenszel test, showing p-value under the additive model, meta-analysis using the adjusted odds ratio and standard error of the point estimate, accompanied by p-het and I-squared index.

SNP	Minor allele	MAF Cases	MAF Control	p-CMH	OR (95% CI)	p-meta	I^2^	p-het
rs3753841	G	0.35	0.33	**0.009**	1.31 (1.1–1.6)	**0.011**	0%	0.62
rs1015213	A	0.08	0.06	**0.004**	1.61 (1.1–2.3)	**0.011**	35.1%	0.21
rs11024102	G	0.36	0.36	0.035	1.25 (1.0–1.5)	0.067	35.1%	0.21
rs3788317	A	0.20	0.21	0.999	0.99 (0.8–1.3)	0.936	0%	0.65

P-value <0.013 is considered significant. MAF: minor allele frequency, I^2^: measures heterogeneity, p-het: p-value for heterogeneity.

**Figure 2:**
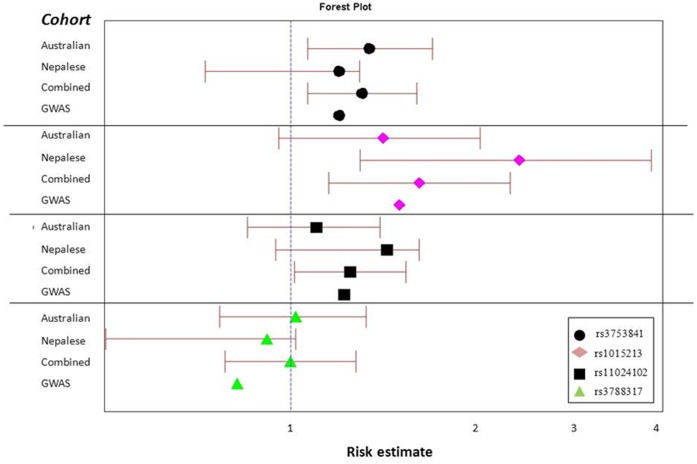
Forest plot showing the odds ratio and 95% CI of the four typed SNPs in the Australian, the Nepalese, the combined analysis and the meta-analysis results of previous GWAS. The risk estimate axis ranges from 0.5 to 4.0.

The differences in the effect size of Australian, Nepalese, combined cohorts and the results from previous GWAS are presented in Figure 2, showing that the direction of association of these three SNPs is the same as the previous GWAS report. SNP rs3788317 did not reveal a statistically significant association with PACG in any analysis.

## Discussion

The molecular mechanisms leading to PACG are poorly understood. In this study we sought to replicate the top ranked SNPs from a recent GWAS of predominantly Asian patients. Our results support the association of three loci (rs3753841, rs1015213, and 11024102) in our cohorts, and demonstrate that carriers of the minor alleles of these SNPs are approximately 1.5 times more likely to develop PACG. The effect size in our Australian cohort is similar to the findings reported in the small UK cohort included in the initial GWAS study.

SNP rs3753841 is located in the *COL11A1* gene (OMIM 120280). It is a missense mutation, situated in a coding region of the gene g.199135C>T (p.Pro1284Leu). The previous GWAS showed the minor allele to be the risk with meta-analysis p-value of 9.22×10^−10^ (OR 1.20). The variant is predicted to be benign and tolerated by PolyPhen2 or SIFT, respectively. This suggests that this variant is unlikely to be the causative allele despite being a non-synonymous change, but it is likely tagging another functional variant that is responsible for this disease.

SNPs rs11024102, and rs3788317 are situated in an intron of the *PLEKHA7* (OMIM: 612686) and *TXNRD2* (OMIM: 606448) genes respectively. SNP rs11024102 met the criteria for genome wide significance in the GWAS (meta-analysis p-value of 5.33×10^−12^, OR 1.22). Our study shows a suggestive association of this SNP. Although it did not reach statistical significance, our finding supports the previous GWAS result. In addition, rs3788317 did not reach genome wide significance in the reported GWAS with meta-analysis p-value of 1.73×10^−7^ (OR 0.82), and it also failed to show association in our study. The inconsistency in the direction of this SNP within our study cohorts and with the previous GWAS study makes rs3788317 unlikely to be a true PACG SNP. We also report that the SNP rs1015213 showed replication in our cohorts. Although both rs11024102 and rs1015213 show a moderate degree of heterogeneity, this was not significant and thus does not affect the conclusions.

The main limitation of this study is the small sample size. However, the effect sizes are in the same direction as previously reported. We considered the cohort underpowered to detect association at rs1015213, however, the observed odds ratio was greater than expected and a significant result was detected at this SNP as well as at the more common SNPs. It is now necessary to investigate how the candidate genes at each of these loci affect the pathogenicity of this blinding disease. In conclusion, this is the first study to replicate findings from a recent genome-wide study, and further confirms the role of *PLEKHA7* and *COL11A1* as candidate genes for PACG. Further analysis is also required to identify how rs1015213 contributes to the disease.
